# Cytoskeletal Transport, Reorganization, and Fusion Regulation in Mast Cell-Stimulus Secretion Coupling

**DOI:** 10.3389/fcell.2021.652077

**Published:** 2021-03-16

**Authors:** Gaël Ménasché, Cyril Longé, Manuela Bratti, Ulrich Blank

**Affiliations:** ^1^Laboratory of Molecular Basis of Altered Immune Homeostasis, Imagine Institute, INSERM UMR 1163, Université de Paris, Paris, France; ^2^Centre de Recherche sur l’Inflammation, INSERM UMR 1149, CNRS ERL8252, Faculté de Médecine site Bichat, Université de Paris, Paris, France; ^3^Laboratoire d’Excellence Inflamex, Université de Paris, Paris, France

**Keywords:** mast cells, signaling, cytoskeleton, actin, microtubule, degranulation, secretory granule transport, secretory granule fusion

## Abstract

Mast cells are well known for their role in allergies and many chronic inflammatory diseases. They release upon stimulation, e.g., via the IgE receptor, numerous bioactive compounds from cytoplasmic secretory granules. The regulation of granule secretion and its interaction with the cytoskeleton and transport mechanisms has only recently begun to be understood. These studies have provided new insight into the interaction between the secretory machinery and cytoskeletal elements in the regulation of the degranulation process. They suggest a tight coupling of these two systems, implying a series of specific signaling effectors and adaptor molecules. Here we review recent knowledge describing the signaling events regulating cytoskeletal reorganization and secretory granule transport machinery in conjunction with the membrane fusion machinery that occur during mast cell degranulation. The new insight into MC biology offers novel strategies to treat human allergic and inflammatory diseases targeting the late steps that affect harmful release from granular stores leaving regulatory cytokine secretion intact.

## Introduction

Mast cells (MC) are granulated cells of the hematopoietic lineage in tissues that localize in large numbers, especially under epithelial and mucosal surfaces exposed to the external environment such as the skin, the airways, and the intestine. This widespread distribution throughout the body attributes them the role of sentinel cells at the interface between innate and adaptive immunity (Beghdadi et al., 2011; Galli et al., 2020). Despite their undeniable role in the regulation of immune responses, MC are best-known as effector cells of allergic diseases after stimulation through their high-affinity IgE receptors (FcεRI). Within minutes after the crosslinking of FcεRI-bound IgE by a specific antigen/allergen, the MC degranulate and release a variety of inflammatory mediators contained in secretory granules (SG) including proteases, proteoglycans, lysosomal enzymes such as β-hexosaminidase, and vasoactive amines such as histamine and serotonin. This is followed (within 15 to 30 min) by the synthesis of lipid mediators, such as leukotrienes and prostaglandins, and (after several hours) by the *de novo* synthesis and secretion of cytokines and chemokines that mediate and regulate the inflammatory response (Blank and Rivera, 2004; Blank et al., 2014; Wernersson and Pejler, 2014).

**FIGURE 1:**
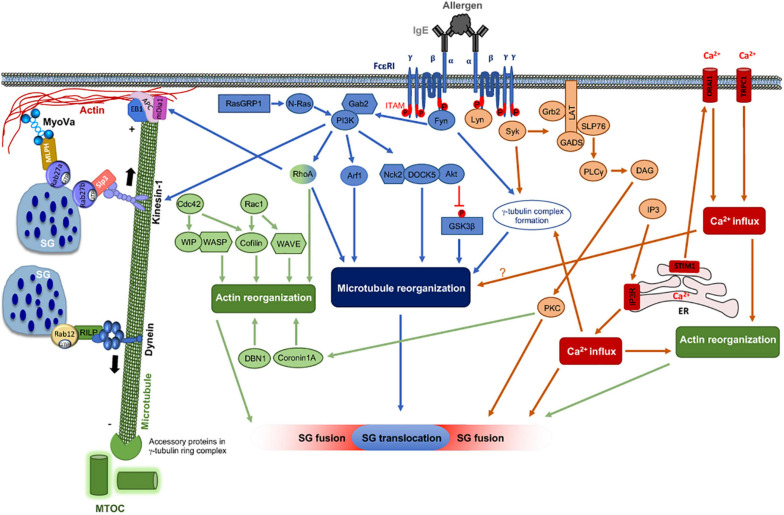
FcεRI signaling pathways involved in cytoskeleton reorganization required for SG transport and fusion. The aggregation of the FcεRI by the IgE-allergen complex leads to phosphorylation of the ITAMs and the PTK (Fyn, Lyn, and Syk). They participate in the phosphorylation of multiple adapter proteins (Grb2, Gab2, SLP76, LAT), which generate additional signalosomes further propagating the signal. FcεRI-mediated signaling can be partitioned into Ca2+-dependent (LAT, SLP76; PLCγ, DAG, IP3, PKC) and Ca2+-independent pathways (Fyn, Gab2, PI3K, RhoA, RasGRP1, Arf1, Nck2, DOCK5, Akt) that mediate microtubule and actin reorganization necessary for SG transport and fusion. Actin dynamics in MC is also regulated by several actors including RhoA, Cdc42, Rac1, WIP, WASP, WAVE, Coronin1A, DBN1, and Cofilin. Microtubule dynamics is regulated by stabilization at the plus-end extremity by the trimeric protein complex EB1/APC/mDia1 and by a crosstalk between Fyn, Syk, and γ-tubulin signaling that may lead to microtubule nucleation at the MTOC or to enhanced non-centrosomal microtubule nucleation. Microtubule-mediated SG retrograde transport on microtubule is mediated by Rab12 that recruits the RILP-dynein complex. SG anterograde transport requires PI3K activation that allows kinesin-1’s accessibility to the cargo receptor Slp3. The SG switch from microtubules to cortical actin is regulated by the Rab27a/Mlph/MyoVa complex. Thin arrows indicate direct effects on signaling cascades, cytoskeleton reorganization, SG translocation, and fusion events.

The earliest signaling event in response to FcεRI crosslinking is the phosphorylation of the immunoreceptor tyrosine-based activation motifs (ITAM) in the cytoplasmic tails of FcεRIβ and disulfide-linked FcεRIγ subunits and the activation of two Src-family protein tyrosine kinases (PTK) Fyn and Lyn followed by recruitment of the PTK Syk to the phosphorylated FcεRIγ ITAM (Rivera et al., 2008; Metcalfe et al., 2009). Activated PTK (in particular Fyn and Syk) then induce the phosphorylation of multiple intracellular adaptor proteins including growth-factor-receptor bound protein 2 (Grb2), Grb2-related adaptor protein (Gads), Grb2-associated binding protein 2 (Gab2), SH2 domain-containing leukocyte phosphoprotein of 76 kDa (SLP-76), and the transmembrane adapter protein linker for activation of T cells (LAT) (Alvarez-Errico et al., 2009). Phosphorylated LAT then enables the plasma membrane (PM) recruitment and activation of phospholipase Cγ (PLCγ) generating diacylglycerol (DAG) and inositol 1,4,5-trisphosphate (IP3) that activate, respectively, protein kinase C (PKC) and Ca2+ influx signaling (Rivera et al., 2008; Metcalfe et al., 2009). Signaling is further enhanced by the TEC family kinase BTK recruited to the PM via phosphatidylinositol (3,4,5)-trisphosphate (PIP3) generated by Gab2 activated phosphatidyl inositol-3 kinase (PI3K) (Metcalfe et al., 2009; Zorn et al., 2018). The release of Ca2+ from the endoplasmic reticulum (ER) induces the stromal interaction molecule 1 (STIM-1) recruitment to the Ca2+ release activated channel (CRAC) Ca2+ release-activated Ca2+ channel protein1 (ORAI1) leading to extracellular Ca2+ influx (Ma and Beaven, 2009; Holowka et al., 2012). Additionally, the transient potential Ca2+ channel 1 (TRPC1) amplifies the Ca2+ influx across the PM leading to an enhancement of the free cytoplasmic Ca2+ concentration, propagating further signaling events (Figure 1; Suzuki et al., 2010).

Early signal transduction downstream of FcεRI is accompanied by several changes in cell morphology based on cytoskeleton reorganization allowing MC degranulation (Draber et al., 2012). Several lines of evidence have shown that the Ca2+ response is crucial to regulate actin remodeling (Koffer et al., 1990) and the later stages of SG fusion with the PM by controlling the soluble N-ethylmaleimide sensitive fusion (NSF) attachment protein receptor (SNARE) complex formation (Blank and Rivera, 2004; Holowka et al., 2012). Gab2 phosphorylation by Fyn has been shown to be critical for the recruitment and activation of phosphatidyl inositol-3 kinase (PI3K) and the small GTPase Ras homology family member A (RhoA), which are required for microtubule formation and SG translocation in a Ca2+-independent manner (Figure 1; Gu et al., 2001; Parravicini et al., 2002; Nishida et al., 2005; Nishida et al., 2011).

Thus, the degranulation process requires the extensive reorganization of the cytoskeleton associated with membrane ruffling and cell spreading, the anterograde movement of SG and their fusion with the PM that leads to the release of inflammatory mediators. Research in recent years on the interplay between the secretory and cytoskeletal machinery that occurs during MC degranulation has highlighted complex processes that are tightly regulated by proximal signaling events downstream of the FcεRI. In this review, we will discuss the molecular processes that regulate stimulus-secretion coupling during MC degranulation highlighting molecular events that regulate the cytoskeleton and transport machinery in conjunction with the membrane fusion machinery.

## Microtubule Dynamics During MC Degranulation

### Microtubule Organization and Dynamics

MC activation by FcεRI aggregation induces increased formation of microtubules leading to microtubule-containing PM protrusions (Nishida et al., 2005; Draber et al., 2012). Microtubules are hollow tubular structures constituted of heterodimers of globular α- and β-tubulin subunits assembled into 13 linear protofilaments. New microtubules are nucleated from a central Microtubule Organizing Center (MTOC) also called centrosome. The minus-ends are capped and anchored to the MTOC with a slow-growing activity exposing α-tubulin subunits while the plus-ends generally are localized to the cell periphery with a fast-growing activity exposing β-tubulin subunits. Hence, microtubules are polarized. At the minus-end, γ-tubulin contributes to microtubule nucleation and stabilization by associating with γ-tubulin complex proteins (GCPs) to form a ring complex named the γ-tubulin ring complex (γ-TuRC) (Oakley et al., 2015). Microtubules undergo phases of growth, pause, and shrinkage, separated by rescue (transition from depolymerization to growth) or catastrophe (transition from growth to depolymerization) events. This microtubule behavior was called “dynamic instability” (Desai and Mitchison, 1997) and was observed *in vitro* and *in vivo* (Schulze and Kirschner, 1986; Burbank et al., 2006). Initiation of the microtubule polymerization requires addition of GTP-bound tubulin subunits at the plus-end of microtubules. Growing microtubule sheets create a cap of GTP-tubulin with stabilizing properties. Cap loss induces rapid depolymerization of microtubules. Microtubule dynamics is also coordinated by external regulators such as stabilizing factors (microtubules plus-end tracking proteins [+Tips], microtubule-associated proteins [MAPs], minus-end capping proteins) and destabilizing factors (depolymerizing kinesins, stathmin and severing proteins) (Akhmanova and Steinmetz, 2015).

### Modulation of Microtubules by Drugs

Microtubule assembly is susceptible to drugs. Taxol is a natural product, and its derivatives induce microtubule assembly by stabilizing microtubules whereas nocodazole, colchicine, vinblastine, and vincristine destabilize microtubules. In MC, treatment with nocodazole and taxol suppressed FcεRI-mediated degranulation and translocation of SG to the PM demonstrating the role of tubulin dynamics in MC degranulation (Nielsen and Johansen, 1986; Martin-Verdeaux et al., 2003; Smith et al., 2003). More recently, miltefosine, a derivative of plasmalogen phospholipids used to treat MC-driven diseases, was described to inhibit the formation of microtubule protrusions on activated MC through the inhibition of DAG-regulated conventional PKC activity (Rubikova et al., 2018).

### Signaling Events Downstream FcεRI Regulating Microtubule Dynamics

Genetic studies in mice were employed to dissect more precisely the molecular mechanisms required for cytoskeletal rearrangement in the degranulation process. It was reported that Fyn-deficient bone marrow-derived MC (BMMC) are defective in degranulation, albeit the Ca2+ influx was intact (Parravicini et al., 2002). Further analysis showed that microtubule formation was impaired in Fyn- and Gab2-deficient BMMC upon FcεRI activation (Nishida et al., 2005). In addition, activation of the small GTPase RhoA, known to regulate cytoskeletal reorganization, was significantly decreased in Gab2-deficient BMMC after FcεRI stimulation (Nishida et al., 2005). Thus, these reports identified a Fyn/Gab2/RhoA proximal FcεRI signaling pathway required for microtubule-dependent SG translocation to the PM (Figure 1). Inhibitors targeting PI3K and genetic inactivation of the p110δ catalytic subunit of PI3K also abrogated SG translocation (Barker et al., 1995; Ali et al., 2004). Activation of PI3K depends on recruitment of its p85 subunit to Gab2 (Gu et al., 2001) and knock-in mice expressing Gab2 mutated for the PI3K binding site were deficient in SG translocation (Nishida et al., 2011). This study also identified the small GTPase, ADP-ribosylation factor 1 (ARF1), as the downstream target of PI3K involved in SG translocation. ARF1 activation depends on Fyn, Gab2, and the Gab2 interaction with PI3K highlighting that the Fyn/Gab2/PI3K/ARF1 signaling pathway is required for FcεRI-mediated SG translocation (Figure 1; Nishida et al., 2011). By contrast this pathway controlling microtubule formation and SG translocation was not required for Ca2+ influx and F-actin disassembly (Figure 1). Another downstream target of PI3K is DOCK5, an atypical guanine nucleotide exchange factor (GEF) for Rac that was shown to regulate microtubule dynamics during MC degranulation independently of its Rac GEF activity (Ogawa et al., 2014). DOCK5 interacts with Nck2 and Akt promoting the phosphorylation and subsequent inactivation of the serine/threonine kinase GSK3β, which negatively regulates microtubule dynamics and MC degranulation (Figure 1; Zhou and Snider, 2005; Ogawa et al., 2014). The Ras guanyl nucleotide-releasing protein 1 (RasGRP1), in parallel to Gab2, also participated to the activation of RhoA and PI3K through the activation of N-Ras (Figure 1; Liu et al., 2007). Genetic inactivation of RasGRP1 was associated in MC with a profound defect in microtubule formation and SG translocation to the PM (Liu et al., 2007).

The formation of microtubules upon FcεRI stimulation could potentially require (i) stabilization of the plus-end extremity of the microtubules, (ii) regulation of the microtubule nucleation at the minus-end of microtubules at the MTOC, or alternatively (iii) enhancement of non-centrosomal microtubule nucleation. Several signaling molecules involved in FcεRI-mediated microtubule reorganization could play a role in these three ways of microtubule nucleation. Activated RhoA was found to regulate microtubule formation upon FcεRI stimulation downstream of Fyn/Gab2 (Nishida et al., 2005). It could act by stabilizing the plus-end extremity of microtubules through the recruitment of mammalian diaphanous-related formin 1 (mDia1) promoting its binding to microtubule plus-end tracking proteins (+Tips), end-binding protein 1 (EB1), and adenomatous polyposis coli (APC) (Palazzo et al., 2001; Wen et al., 2004; Wojnacki et al., 2014). This trimeric protein complex functions as a microtubule plus cap, which prevents heterodimer exchange, thereby stabilizing microtubules at the PM (Figure 1). In addition, several evidences show a crosstalk between Fyn, Syk, and γ-tubulin-signaling complexes. This could lead, in activated MC, to microtubule nucleation at the MTOC or to enhanced non-centrosomal microtubule nucleation (Sulimenko et al., 2006). Work from the same team showed that in differentiating P19 embryonal carcinoma cells, γ-tubulin was recruited to the PM through a direct interaction with PI3K enabling non-centrosomal microtubule nucleation (Macurek et al., 2008). Moreover, microtubule nucleation in BMMC has recently been shown to involve proteins that are associated with γ-tubulin, such as p21-activated kinase interacting exchange factor β (βPIX, also known as Rho guanine exchange factor 7) and G protein-coupled receptor kinase-interacting protein 1 (GIT1) (Sulimenko et al., 2015). βPIX and GIT1, in association with centrosomes, have opposite function on microtubule nucleation, affecting either negatively or positively microtubule growth. Surprisingly, the interaction between GIT1 and γ-tubulin was Ca2+-dependent, suggesting a role of Ca2+ leading to microtubule nucleation in activated MC (Figure 1; Sulimenko et al., 2015). Recently, protein tyrosine phosphatases were also found to be important for the regulation of microtubule nucleation. Indeed, the Src homology 2 domain-containing protein tyrosine phosphatase 1 (SHP-1) is present in complexes containing γ-tubulin, GCP2, GCP4, and Syk and modulates negatively microtubule nucleation from the centrosomes of BMMC (Klebanovych et al., 2019).

While several studies showed the role of Ca2+ in the regulation of microtubule remodeling (Hajkova et al., 2011; Cruse et al., 2013; Sulimenko et al., 2015) another study that used Ca2+-free medium or medium containing EGTA with or without xestospongin (IP3 receptor inhibitor) found that these conditions did not inhibit FcεRI-induced microtubule formation (Nishida et al., 2005). This discrepancy may be attributable to differences in cell activation and various methods of sample preparation. Another explanation is that initial steps of microtubule formation and granule displacement could be Ca2+-independent, whereas later steps of MC activation and protrusion formation could be dependent on calcium influx (Draber et al., 2012). A truncated splice variant of the FcεRIβ subunit, t-FcεRIβ, known to bind to Gab2, Fyn, and calmodulin, could act to propagate Ca2+ signaling for microtubule nucleation in human LAD-2 MC. After MC activation t-FcεRIβ localized to the Golgi in close contact with the pericentrosomal region and hence may involve the Golgi complex to regulate Ca2+-dependent microtubule formation required for MC degranulation (Cruse et al., 2013). STIM-1 is one of the key components that regulates the influx of extracellular Ca2+ mediated by the opening of store-operated channels (SOCs). Interestingly, STIM-1 was also described as a microtubule-tracking protein that interacts with EB1 (Grigoriev et al., 2008). Knockdown approaches identified STIM1 as being required for the formation of microtubule protrusions (Hajkova et al., 2011; Cruse et al., 2013). By contrast, intact microtubules were not required for STIM-1 aggregation and recruitment beneath the PM to support opening of CRAC channels.

Although the complexity of signaling leading to the regulation of microtubule dynamics upon MC activation starts to be described (Figure 1), further studies are still required to understand the full complexity of the coordination of microtubule dynamics and secretion regulation in MC.

## Actin Dynamics During MC Degranulation

### Actin Organization and Regulation

Actin is highly abundant, comprising 1 to 5% of the total cellular proteins. It exists as a monomer called G-actin (globular) that can polymerize into long helical F-actin (filamentous) microfilaments (Pollard, 2016). They can be held together by crosslinking proteins to form actin bundles and networks (e.g., α-actinin, fimbrin, fascin, filamin, spectrin, dystrophin) or branch using specific effectors (e.g., actin-related proteins 2/3, [Arp2/3] complex) (Broderick and Winder, 2005; Zhou et al., 2010; Liem, 2016; Pollard, 2016; Machnicka et al., 2019). Actin interacts with a substantial number of proteins that contribute to maintain a pool of actin monomers, initiate polymerization, constrain the length of actin microfilaments, regulate their assembly and turnover, or crosslink them into bundles or networks (dos Remedios et al., 2003; Pollard, 2016). The actin cytoskeleton consists of structurally and biochemically different actin filament arrays. Although still poorly defined, the one attached to the membrane, called cortical actin, is planar forming a web beneath the PM (Svitkina, 2020), while within the cell the actin cytoskeleton is three-dimensional providing the cytosol with gel-like properties (Pollard, 2016). The actin cortex is attached to the membrane involving other proteins of the ERM (Ezrin, Radixin, Moesin) family and the spectrin network, all of which may contribute to signaling functions (Machnicka et al., 2014; Garcia-Ortiz and Serrador, 2020). The actin cytoskeleton provides a framework for cell shape, yet an important characteristic is that it is highly dynamic thereby (i) facilitating the transduction of mechanical signals, (ii) generating forces that allow cell motility, cell division, cytokinesis, and (iii) allow vesicular trafficking and muscle contraction (Svitkina, 2018), (iv) contribute to the formation and maintenance of cell junctions (Zhang et al., 2005), and (v) participate in cell signaling (Draber et al., 2012; Mattila et al., 2016). Actin dynamics is regulated by small GTPases of the Rho family (Rho, Rac, Cdc42) in response to external signals. Rho activates the formation of stress fibers and focal adhesions, Rac enables formation of lamellipodia and membrane ruffles and Cdc42 activates the formation of filopodia (Hall, 1998; Heasman and Ridley, 2008). Rho family GTPases act as molecular switches that enable either directly or indirectly the activation of multiple downstream signaling effectors of actin dynamics including kinases (p21-activated kinase [PAK]); Rho-associated coiled-coil kinase (ROCK); LIM-motif containing kinase (LIMK); myosin light chain phosphatase (MLCP); myosin light chain kinase (MLCK); as well as nucleation and branching promoting factors (mDia); Wiskott-Aldrich syndrome protein (WASP); Wiskott-Aldrich syndrome protein-family verprolin homologous protein (WAVE); Arp2/3 complex (Heasman and Ridley, 2008; Draber et al., 2012).

### Actin Dynamics in MC

MC signaling is accompanied by important changes in the actin cytoskeleton. Initial data showed that FcεRI-mediated stimulation in rat basophilic leukemia (RBL) MC promotes a decrease in cellular F-actin content during the first 10–30 s followed by a rapid increase within 1 min. This coincided with the transformation of the surface topography to a less dense actin cortex and formation of lamellar actin ruffles at the cell surface as well as cell spreading (Pfeiffer et al., 1985; Frigeri and Apgar, 1999; Wilson et al., 2016). This was confirmed in cultured primary and *ex vivo* isolated MC (Pendleton and Koffer, 2001; Tumova et al., 2010). Additional data showed that during stimulation the cortical actin becomes fragmented and disassembled (Koffer et al., 1990; Narasimhan et al., 1990) in a Ca2+ and calmodulin-dependent manner strongly correlating with secretion (Sullivan et al., 2000; Nishida et al., 2005). This supported earlier data in other cells (Orci et al., 1972; Cheek and Burgoyne, 1986) indicating that the actin web could represent a physical barrier for secretion with its disassembly representing a terminal step in exocytosis. In agreement with the assumption that actin remodeling is necessary for secretion to occur, addition to MC of Jasplakinolide, a drug that stabilizes F-actin polymers, inhibited MC degranulation (Nishida et al., 2005; Wilson et al., 2016), while Cytochalasin D and Latrunculin A, which inhibit actin polymerization thereby disrupting microfilaments, enhanced MC secretion (Narasimhan et al., 1990; Pierini et al., 1997; Frigeri and Apgar, 1999; Martin-Verdeaux et al., 2003).

Yet, data accumulated to date show that the signal-induced changes in the actin cytoskeleton are far more complex relating to both early and late signaling events. Studies with actin disrupting agents showed that they promoted an increased and prolonged phosphorylation response including that of the FcεRI β and γ subunits (Frigeri and Apgar, 1999; Holowka et al., 2000; Tolarova et al., 2004; Torigoe et al., 2004) and an increase in intracellular Ca2+ levels (Oka et al., 2002). Fluorescence localization microscopy and pair-correlation analysis indicated that this was associated with an enhanced colocalization of IgE-FcεRI, Lyn, and a lipid anchor probe of Lyn in ordered lipid regions. This supports that the actin cytoskeleton might regulate this functional interaction by influencing the organization of membrane lipids (Shelby et al., 2016). In addition to its barrier function, the actin cortex also acts as a carrier for myosin V actin motors to capture and transport vesicles to membrane fusion sites, thereby playing a role in corraling and docking SG at the PM (Elstak et al., 2011; Wollman and Meyer, 2012; Singh et al., 2013). Based on these evidences, MC activation was clearly associated with signaling of actin remodeling.

### Signaling Events Downstream FcεRI Regulating Actin Dynamics

Early studies showed that the GTPase Rho participated in actin polymerization, while Rac favored reorientation of microfilaments, and both proteins were implicated in secretion in rat peritoneal MC (Norman et al., 1994, 1996; Price et al., 1995). In RBL MC, activated Cdc42 participated in the formation of cell adhesions while Rac1 played a role in FcεRI-mediated membrane ruffling. Expression of trans-dominant inhibitory forms of both Cdc42 or Rac1 significantly inhibited antigen-induced degranulation (Guillemot et al., 1997). Recent studies using specific inhibitors confirmed that Rac proteins triggered F-actin-mediated protrusions and flattening of the cell periphery to create an active degranulation zone, whereas RhoA participated in ruffle formation also controlling granule motility (Sheshachalam et al., 2017). The involved actin remodeling plays a role in FcεRI early signaling and Ca2+ responses possibly via by its effect on the activation of cofilin (see also below) (Oka et al., 2004; Ang et al., 2016). Indeed, when receptors were desensitized through repeated stimulation with increasing doses of antigen, dynamic reorganization of the actin cytoskeleton is inhibited. This is associated with an inhibition of the cofilin dephosphorylation/phosphorylation cycle regulating actin dynamics (Ang et al., 2016). Regarding downstream signaling of Rho GTPases, genetic knockout (KO) and knockdown (KD) as well as inhibitor approaches identified a Pak1 kinase-dependent interaction with protein phosphatase 2 (PP2A) promoting dephosphorylation of Thr567 of Ezrin/Radixin/Moesin (ERM) proteins. This uncouples the PM from the actin cytoskeleton prior to F-actin clearing and degranulation. Absence of this axis led to defective F-actin rearrangement, impaired degranulation, and systemic histamine release (Staser et al., 2013). Other important downstream targets of Rho GTPases are WASP (downstream of Cdc42) and Wave (downstream of Rac) proteins promoting the association with the Arp2/3 complex to create a nucleation core for actin branching (Figure 1; Moller et al., 2019). In WASP-deficient BMMC actin polymerization, cell spreading, formation of ruffles, and degranulation was inhibited after FcεRI stimulation (Pivniouk et al., 2003). Likewise, although some pleiotropic actions including on early signaling responses were observed, the Wiskott-Aldrich syndrome protein interacting protein (WIP), which holds WASP in an inactive state, was found to play a role in degranulation and generation of actin filaments in FcεRI-stimulated BMMC (Figure 1; Kettner et al., 2004). All these data strongly indicate that Rho GTPase family members and their effectors have a major role in organization of microfilaments and degranulation responses in activated MC. Drebrin (DBN) is another actin-associated protein able to crosslink F-actin bundles, thereby stabilizing the actin network (Figure 1). In DBN1-KO MC, histamine and *in vivo* passive systemic anaphylactic responses are inhibited, indicating a positive regulatory role (Law et al., 2015). Further analysis showed that DBN1-KO MC exhibited defects in actin organization with actin bundles accumulating in the cytoplasm and a delay in F-actin clearance in stimulated cells. The effect on degranulation could be rescued by the actin depolymerizing drug Latrunculin B. The DBN1-KO MC also showed a defect in intracellular Ca2+ influxes after stimulation, as was also observed in WASP- (Pivniouk et al., 2003) and WIP-KO MC (Kettner et al., 2004), again supporting a role of the actin network in Ca2+ responses. In contrast, the actin-binding protein Coronin1A, which binds to F-actin via its WD40 repeat domain, was reported to negatively regulate exocytosis (Figure 1; Foger et al., 2011). In its absence degranulation was enhanced mimicking the effects of actin depolymerizing drugs, whereas cytokine secretion (but not production) was inhibited. FcεRI stimulation phosphorylated Coronin1A on the PKC substrate Ser2 promoting its relocation from the cortex to the cytoplasm and cortical F-actin disassembly, hence providing genetic evidence for its function as a physical barrier. The positive action on cytokine secretion of Coronin1A points to a role of F-actin cytoskeleton remodeling in the egress of transport vesicles from the trans-Golgi. A recent study points to the contribution of the actin-severing protein cofilin in cortical actin depolymerization/polymerization in MC (Suzuki et al., 2021). Following receptor crosslinking with a hapten-antigen, cofilin becomes rapidly dephosphorylated promoting cortical actin disassembly. When disaggregating receptors with the free hapten, cofilin rephosphorylates, thereby restoring the cortical actin barrier. The actin polymerizing effector mDia1, when activated through chemokine receptors, but not by FcεRI, was shown to participate in the buildup of pericentral actin clusters that converge the SG to the cell center, preventing them to undergo secretion (Klein et al., 2019). The formation of these clusters presents therefore a mechanism to favor migration over secretion in cells stimulated via chemokine receptors. Interestingly, mDia1 has been reported to generate actin filaments that are resistant to the action of cofilin (Mizuno et al., 2018). The filaments generated by this formin may also play a role in the terminal trafficking steps close to the cortex that could depend on actin as shown in pancreatic acinar cells (Geron et al., 2013). However, presently this has not been investigated in MC.

### Actin Facilitating SG Secretion

Although it was proposed that the cortical actin ring may act as a physical barrier in MC (Koffer et al., 1990), observations in other cells are also compatible with the assumption that microfilaments provide tracks for myosin-dependent SG mobility (Oheim and Stuhmer, 2000). This process may involve actin coating of SG as shown in pancreatic acinar cells favored by the release of Rab3D GTPase from them (Valentijn et al., 1996, 1999). Actin coating and myosin may also serve as mechanical forces for the expulsion of granular content and/or eventually stabilize fusing granules (Nightingale et al., 2012). Although it was found that Rab3D can regulate SG fusion in MC (Roa et al., 1997; Tuvim et al., 1999), its possible involvement in actin granule coating has not been investigated in these cells, yet. The Rab GTPase Rab27a has also been proposed to play a role in coupling SG to the actin cytoskeleton (Fukuda, 2013). In MC, Rab27a regulates cortical actin integrity limiting secretion by restricting the access of SG to the PM. It functions likely together with its interacting partners Melanophilin and Myosin Va as mice deficient in these proteins similarly show a slightly hypersecretory phenotype (Figure 1 and Table 1; Singh et al., 2013). Comparison of the secretory phenotype of Rab27b-KO and Rab27a/b double KO indicated that Rab27a may also play a positive role acting in concert with Rab27b to facilitate degranulation (Mizuno et al., 2007; Singh et al., 2013). This involves the switching of SG from a microtubule-dependent movement to an F-actin-dependent movement, finally allowing to corral and dock motile SG at the PM in conjunction with Munc13-4 (Elstak et al., 2011; Singh et al., 2013). As shown in neutrophils, Rab27a action on the actin cytoskeleton may also involve another effector of the Synaptotagmin-like protein (Slp) family such as Slp1 shown to bind to the PM via its C2A domain. This promotes the interaction with the Rho GAP Gem-interacting protein (GIMP) leading to the inhibition of RhoA, thereby facilitating actin depolymerization (Johnson et al., 2012; Ramadass and Catz, 2016). Besides Rab27a, Rab11 may represent another Rab protein regulating actin dynamics. Rab11 localizes to recycling endosomes (RE) in MC. Expression of a dominant negative form (S25N) inhibited antigen stimulated exocytosis of vesicles emanating from the RE, which was rescued in the presence of actin depolymerizing drugs. Further mechanistic studies indicated that PM-recruited Rab11 played a role in the regulation of actin depolymerization when activated through its GTPase activating protein p50RhoGAP (Wilson et al., 2016).

**TABLE 1:** Proteins implicated in SG fusion.

Protein (Hu/mo gene name)	Functional domains	Role in MC exocytosis
SNAP-23 = Synaptosomal-associated protein 23 (*SNAP23/Snap23*)	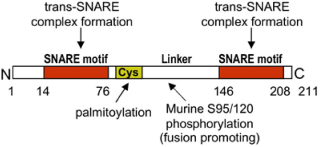	PM-localized t-SNARE; enhanced trans-SNARE complex formation and cytoplasmic relocation along degranulation channels (SG and SG-PM fusion) in stimulated MC; Inhibition of stimulated exocytosis in KD MC and after introduction of blocking Abs
STX3 = Syntaxin 3 (*STX3/Stx3*)	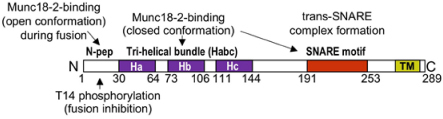	SG-localized t-SNARE; enhanced trans-SNARE complex formation and PM relocation (SG-SG and SG-PM fusion) in stimulated MC; inhibition of stimulated exocytosis in KO/KD MC
STX4 = Syntaxin 4 (*STX4/Stx4*)	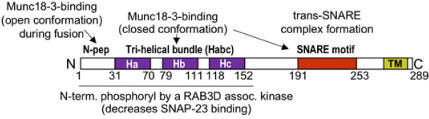	PM-localized t-SNARE; enhanced trans-SNARE complex formation upon stimulation (role in SG-PM fusion?); no inhibition of stimulated exocytosis in KO MC; partial inhibition in KD MC
STX11 = Syntaxin 11 (*STX11/Stx11*)	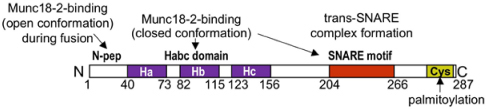	Expression upregulated upon IgE/Ag and LPS stimulation; no inhibition of stimulated exocytosis in KO MC; plays a role in lytic granule exocytosis in NK and T cells
VAMP8 = Vesicle-associated membrane protein-8, also endobrevin (*VAMP8/Vamp8*)	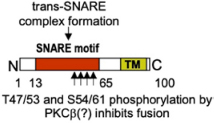	Endosomal, lysosomal and SG-localized v-SNARE; enhanced trans-SNARE complex formation in stimulated MC (role in SG and SG-PM fusion); partial inhibit of stimulated exocytosis in KO and KD MC; one manuscript reports specific effect on β-hexosaminidase but not histamine secretion
VAMP7 = vesicle-associated membrane protein 7, also Tetanus Insensitive Ti-VAMP (*VAMP7/Vamp7*)	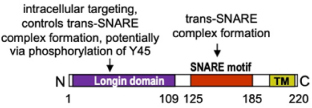	Punctuate staining pattern in resting hu MC (SG?), PM relocation in stimulated primary hu MC; enhanced trans-SNARE complexes upon stimulation in hu MC; inhibition of stimulated exocytosis In KD MC and after introduction of blocking Abs
VAMP2 = vesicle associated membrane protein 2, also Synaptobrevin-2 (*VAMP2* or *SYB2/Vamp2* or *Syb2*)	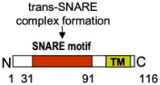	Transfected (but not endogenous) fluorescent VAMP2 showed cytoplasmic (SG?) staining; PM translocation in transfected, stimulated RBL cells, enhanced trans-SNARE complexes in stimulated VAMP8 KO but not in WT BMMC; no inhibition of stimulated exocytosis in KO BMMC, inhibition with blocking Abs in RBL but not hu MC
SYT2 = Synaptotagmin-2 (*SYT2/Syt2*)	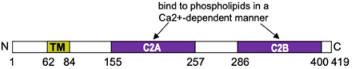	SG-localized Ca2+ sensor; inhibition of stimulated exocytosis in KO MC
CPLX2 = Complexin 2, also Complexin II or Synaphin-1 (*CPLX2/Cplx2*)	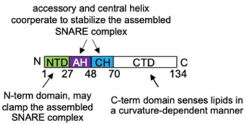	Punctuate cytoplasmic staining; PM translocation in stimulated RBL cells, pulldown assay reveals binding to an assembled STX3 (not STX4)/SNAP-23/VAMP2 or VAMP8 SNARE complex; stimulated exocytosis is inhibited in KD RBL cells, Ca2+ titration experiments show that CPLX2 increases Ca2+ sensitivity
Munc13-4 = mammalian uncoordinated protein 13-4, also protein unc-13 homolog D (*UNC13D/unc13d*)	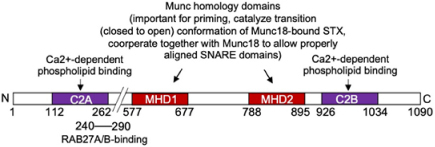	Interacts with SG localized Rab27a or b? through a non-canonical motif, the Rab27/Munc13-4 complex is necessary to dock SG at the PM; substantial inhibition of stimulated exocytosis in KO MC; in neuronal cells Munc13 homologues promote disassembly of the closed Munc18/STX complex; assures together with Munc18 proper parallel SNARE zippering
DOC2α = Double C2-like domain-containing protein alpha (*DOC2A/Doc2a*)	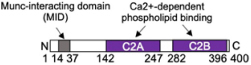	Interacts with Munc13-4 on SG via N-terminal MID and C-terminal C2B domain, colocalizes with SG, partial inhibition of stimulated exocytosis in KD and KO MC
Munc18-2/Munc18b = mammalian uncoordinated protein 18-2/18b, also syntaxin binding protein 2 (STXBP2) or protein unc-18 homolog 2/b (*STXBP2/Stxbp2*)	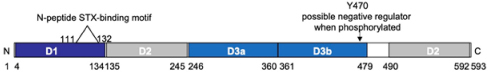	Interacts with SG-localized STX3, Munc18 has two binding modes: binding in closed conformation to a STX SNARE blocks SNARE zippering; binding in an open conformation to STX SNARE (implicating N-terminal peptide) enables zippering; assures together with Munc13-4 proper parallel SNARE zippering
SCAMP2 = Secretory carrier-associated membrane protein 2 (*SCAMP2/Scamp2*)	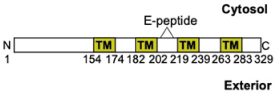	A tetraspanin that may participate in organizing the phospholipid composition for fusion pore formation by coupling Arf6-stimulated PLD generating PIP2; implicates a peptide (E-peptide) as its expression inhibits stimulated exocytosis in MC
STXBP5 = Syntaxin binding protein 5 or tomosyn-1 or Lethal(2) giant larvae protein homolog 3 (*STXBP5/Stxbp5*)	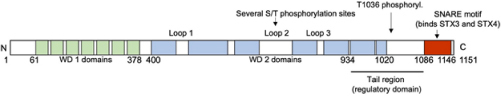	Binds to STX3 and STX4; acts as a fusion clamp as stimulated exocytosis is enhanced in KD MC; STX4 binding decreases and STX3 increases after stimulation in MC; STXBP5 becomes phosphorylated on multiple S/T residues during stimulation promoting STX3 association and STX4 dissociation
Rab3D = Ras-related protein Rab3D (*RAB3D/Rab3d*)	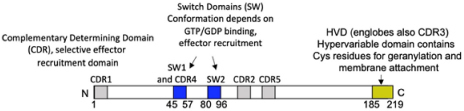	SG-localized; transient PM relocation upon stimulation; “GTP-bound” mutant inhibits exocytosis; no effect in KO MC (compensatory mechanism?); role in actin coating of SG?
RAB5 = Ras-related protein Rab5 (*RAB5/rab5*)	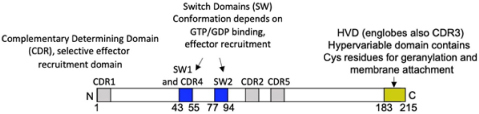	Role in SG maturation mediating SG fusion during biogenesis; role in stimulated exocytosis favoring SG recruitment of SNAP-23 and SG-SG fusion; In KD MC balance is shifted from compound to full exocytosis with SG-PM fusion only
RAB27A = Ras-related protein Rab27A (*RAB27A/Rab27a*)	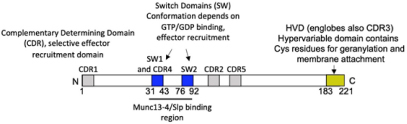	SG-localized; regulates cortical actin integrity; switches SG from microtubule-dependent movement to F-actin-dependent docking; enhanced stimulated exocytosis in KO MC; but facilitates stimulated exocytosis together with Rab27b and Munc13-4 (Rab27a magnifies inhibitory effect of Rab27b on stimulated exocytosis in DKO MC)
RAB27B = Ras-related protein Rab27B (*RAB27B/Rab27b*)	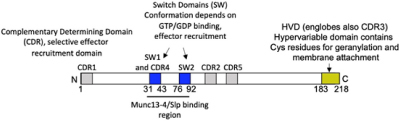	SG-localized; regulates microtubule-dependent movement of SG connecting via Slp3 to the kinesin-1 motor; inhibition of stimulated exocytosis in KO MC, may act partly together with Rab27a as inhibition of stimulated exocytosis is magnified in DKO MC
RAB37 = Ras-related protein Rab37 (*RAB37/Rab37*)	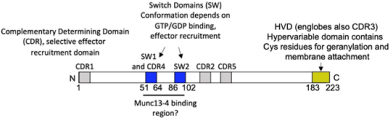	SG-localized, GTP-bound Rab37 interacts with Munc13-4 in a trimeric complex containing Rab27 and Munc13-4; KD MC and transfected GTP-bound Rab37 exhibit hypersecretory phenotypes; Rab37 may counteract the Rab27/Munc13-4-dependent docking/priming step

The possible simultaneous function of the actin cortex as a release barrier and as a carrier for vesicle transport to the PM in the terminal steps in MC secretory signaling has been investigated in more detail with advanced imaging approaches. Using a variety of biosensors (actin, Ca2+, PIP2, N-WASP, Dextran) Wollman and Meyer found that actin remodeling during secretion in RBL MC is tightly coupled to Ca2+ oscillations (Wollman and Meyer, 2012). FcεRI stimulation induced waves of intracellular Ca2+ and PIP2 lipid levels that, in turn, regulate cyclic recruitment of WASP and cortical actin assembly/disassembly. Dextran labeled granules get captured by actin when cortical F-actin levels are high, followed by vesicle passage through the cortex when F-actin starts to disassemble with vesicle fusion at the PM taking place at Ca2+ peak values. Likewise, TIRF image analysis of the cortical actin skeleton in RBL MC revealed a highly orchestrated series of events (Colin-York et al., 2019). Stimulation initially induced cell spreading. Then at the contact interface, the central F-actin network of the cortex exhibiting bright cluster-like actin plaques underwent a symmetry break associated with disassembly of the actin cortex before reassembling again at later time points. These processes appeared intimately linked to the formation of distinct nanoscale F-actin architectures (asters, vortices) that only form in activated cells and were driven by Arp2/3 nucleation. Myosin II motor protein did not colocalize with these patterns, but accumulated in structures around, finally promoting their disassembly supporting an important role in pattern maturation and disassembly. Extrusion of granular content (as measured by appearance of Annexin V staining) was prominent at the contact interface and was closely related to actin dynamics with the dense mesh abruptly forming a small (∼1 μm) circular opening, which remained stable during extrusion. Another study using combined atomic force and laser scanning confocal microscopy in BMMC again showed that stimulation is associated with important changes in F-actin structures with the cell forming lamellipodia and ruffles when adhering to poly-lysine coated surfaces (Deng et al., 2009). As observed in the above study, the cortical F-actin moves to the periphery removing the barrier, thereby favoring permanent fusion as compared to kiss-and-run fusion observed in cells where surfaces were not coated with poly lysine and where actin clearing is much less prominent. The granule extrusion sites appear as craters and are clearly excluded from the F-actin ruffles at the cell surface as observed previously (Martin-Verdeaux et al., 2003).

Taken together, the results accumulated over many years show a tight connection between actin remodeling and secretory functions. How these complex and highly dynamic events are controlled by upstream signaling molecules remains still a puzzle that now, however, can be approached through modern imaging techniques.

## Molecular Mechanisms Regulating SG Trafficking in MC Secretion

**FIGURE 2:**
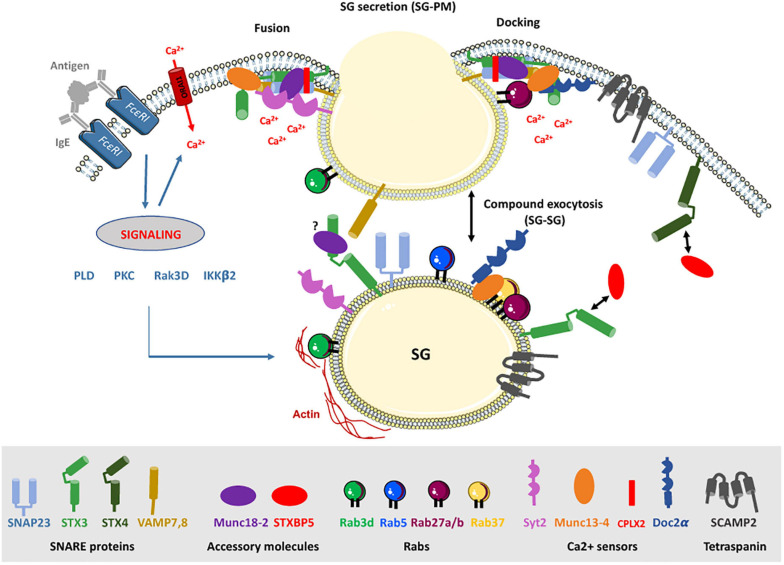
The docking/fusion machinery in MC degranulation. FcεRI aggregation initiates proximal signaling that lead to increases of intracellular Ca2+ levels and the activation of PKC, PLD, Rak3D, and IKKβ2. These signaling events enable SG docking at the PM and their fusion to release granular content. In MC, fusion occurs also between SG, a process known as compound exocytosis, enabling massive release of inflammatory mediators. For simplicity the docking and fusion steps are detailed only at the PM. Note, however, that because of the compound exocytosis mechanisms, many of the molecular effectors are localized on SG eventually redistributing and serving also at the PM after stimulation (see text of Table 1 for further details). In addition, some effectors (e.g., SNAP-23) reversely relocate to SG to serve in the SG fusion process. This process has been shown to be regulated by Rab5 and Ikkβ2. Membrane fusion involves SNARE proteins that lie on opposing membranes. In MC, they include the t-SNARE SNAP-23, STX 3 and 4, and the v-SNAREs VAMP 7 and 8 (see text and Table 1 for further details). Fusion involves formation of a tetrameric trans-SNARE complex that is regulated by numerous accessory proteins allowing SG to dock at the PM or between themselves (not shown) and to facilitate their fusion. Docking involves a complex between Rab27, Munc13-4, and Doc2α and the Ca2+-activated phospholipid binding of Munc13-4 and Doc2α C2 domains. The formation of the docking complex is prevented by Rab37 when bound in a tripartite complex with Rab27 and Munc13-4. SNARE complex formation and fusion is regulated by the concerted action of Munc18-2 and Munc13-4 that allow the proper alignment of SNARE helices and is further assisted by CPLX2 and the Ca2+ sensor Syt2 that triggers fusion at high Ca2+ levels. STXBP5/tomosyn-1, by binding to its STX partner (STX3/4) in a manner regulated by PKCδ, controls the availability of fusion-competent SNARE proteins available for fusion. SCAMP2 may participate in the generation of an appropriate phospholipid composition through the coupling of Arf6-stimulated PLD activity. Rab3D, besides regulating Rak3D activity known to phosphorylate STX4 and inhibit SNAP-23 binding, may have a possible role in actin coating of SG supporting terminal transport and SG content extrusion.

Rab GTPase proteins are well known to regulate and coordinate discrete vesicular trafficking steps along the endocytic and exocytic pathway in all cell types. Rab GTPases function as molecular switches that alternate between the GTP-bound “on” form and the GDP-bound “off” form (Stenmark, 2009). More than 60 Rab family members have been identified from mammalian species and each localizes to a particular membrane compartment. Using a functional screening assay of 44 Rab proteins, 30 of them were identified with a potential role as regulators of MC SG trafficking and exocytosis (Azouz et al., 2012). The Rab27a and Rab27b are both expressed in BMMC and localize to SG (Table 1). The use of single murine KO for Rab27a or Rab27b and the double KO of Rab27a/b demonstrated that the Rab27 family and particularly Rab27b plays a crucial role in MC degranulation (Figure 1; Mizuno et al., 2007). Indeed, as explained above, Rab27a and Rab27b have distinct and opposing roles, Rab27a acts as a negative regulator through its action on actin (Figure 1), whereas both Rab27a and Rab27b act as positive regulators through their interaction with Munc13-4 (Figure 2; Singh et al., 2013). Another Rab GTPase that acts as a negative regulator of MC degranulation is Rab12 described to mediate microtubule-dependent retrograde transport of SG (Efergan et al., 2016). Rab12 transports SG toward the minus-end on microtubules to the perinuclear region upon MC activation by interacting with Rab-interacting lysosomal protein (RILP) within the RILP-dynein complex (Figure 1; Efergan et al., 2016). Indeed, in MC SG were shown to move bidirectionally on the microtubule network (Smith et al., 2003; Nishida et al., 2005; Brochetta et al., 2014). More recently, the plus-end directed microtubule-dependent transport of SG was attributed to the archetypal member of the kinesin superfamily, kinesin-1 (Munoz et al., 2016). Kinesin-1 is composed of two heavy chains (KIF5A, KIF5B, or KIF5C) and two light chains (KLC1, KLC2, KLC3, or KLC4) with KIF5B and KLC1 being the main isoforms present in BMMC. The use of a conditional murine model lacking *Kif5b* in MC demonstrated that kinesin-1 regulates SG transport toward the secretion site during FcεRI activation (Munoz et al., 2016). Upon stimulation, kinesin-1 couples to Slp3 known to interact through its Slp homology domain (SHD) with Rab27b on SG (Figure 1). The stimulation-induced coupling required PI3K activity highlighting that activation regulates kinesin-1 accessibility to the cargo receptor Slp3 (Figure 1). Interestingly, this coupling occurred even in the absence of assembled microtubules in cells treated with nocodazole (Munoz et al., 2016). Rab44, an atypical Rab GTPase, has recently been shown to be involved in MC degranulation and IgE-mediated anaphylaxis (Kadowaki et al., 2020). Rab44 belongs to the family of large Rab proteins that include the founder member CRACR2A and Rab45 (Srikanth et al., 2016). Their carboxy-terminal Rab domain is linked to additional domains, including an EF-hand domain, a coiled-coil domain, and a proline-rich domain that (in CRACR2A) interacts, respectively, with Ca2+, ORAI1/STIM1, and VAV1. In T lymphocytes, CRACR2A is recruited to the immunological synapse through its interaction with VAV-1, where it facilitates CRAC channel function by stabilizing the ORAI1-STIM1 interaction in response to TCR stimulation (Srikanth et al., 2010, 2016). Interestingly, CRACR2A is also able to interact with dynein to regulate endocytic trafficking in a Ca2+ dependent manner (Wang et al., 2019). Although CRACR2A links vesicular trafficking and signaling pathways upon TCR stimulation in T cells, it is not clear whether Rab44 is involved in Ca2+ signaling or other signaling pathways in MC to induce SG secretion.

In addition to Rab GTPases and molecular motors, the mammalian uncoordinated18 (Munc18) isoform 2 (Munc18-2) (Table 1), besides acting as fusion accessory protein, may also act in SG translocation (Brochetta et al., 2014). Treatment with the microtubule-destabilizing drug nocodazole induced redistribution of Munc18-2 from a granular to a diffuse cytosolic location, indicating that its recruitment/docking to SG was dependent on microtubules. Munc18-2 localized to SG, and upon stimulation Munc18-2 was translocated to the periphery into forming lamellipodia along microtubules remaining associated with large (fused) SG (Brochetta et al., 2014). During stimulation, the interaction of Munc18-2 with β-tubulin was downmodulated, indicating a possible dynamic functional interaction with the microtubule cytoskeleton. In agreement, KD of Munc18-2 affected SG translocation and docking at the PM with SG appearing stationary docked along microtubules inside the cell. This suggests that Munc18-2 could dynamically dock SG during microtubular transport, eventually in conjunction with the kinesin1/Slp3/Rab27b transport mechanism described above (Brochetta et al., 2014). In this context, neuronal Munc18-1 was demonstrated to bind to the kinesin-1 adaptor protein, fasciculation, and elongation protein zeta 1 (FEZ1 or zygin) to mediate axonal transport along microtubules, providing a link to microtubule-dependent vesicular transport (Chua et al., 2012).

Although some clues have been obtained how the molecular machinery of SG transport gets linked to signaling pathways downstream of FcεRI, much work remains to be done to understand further the complex signaling pathways of SG trafficking.

## Fusion Machinery in MC Secretion

The terminal step in MC degranulation is the fusion of the SG membrane with the PM to release stored inflammatory mediators into the surrounding environment (Lorentz et al., 2012; Blank et al., 2014). In MC this generally involves, besides the release of soluble mediators, the extrusion of the granular proteoglycan core to which inflammatory compounds are bound via electrostatic interactions (Lawson et al., 1975; Nitta et al., 2009). The change in pH (from acidic to neutral) allows their diffusion into the surrounding environment (Wernersson and Pejler, 2014). MC perform multigranular or compound exocytosis characterized, respectively, by the extrusion of the content of multiple intracellularly fused SG or sequential fusion of SG from the periphery into the interior (Figure 2; Röhlich et al., 1971; Alvarez, de Toledo and Fernandez, 1990). This enables MC to secrete up to 100% of granular content in one single stimulatory event. Under certain conditions of activation MC are also able to perform piecemeal degranulation (PMD) characterized by the gradual emptying of SG content without any evidences for fusion events (Dvorak et al., 1994; Crivellato et al., 2003). Early ultrastructural analysis indicated that this could be due to the budding of intravesicular tubular compartments moving toward the PM (Dvorak et al., 1980; Melo et al., 2005). However, another possibility could be that PMD is the result of transient fusion events also called “kiss-and-run fusion,” which are readily observed in MC (Williams and Webb, 2000). New studies also indicate that MC can change their degranulation pattern from a unit mode to a multigranular/compound mode depending on the type of stimulus (e.g., MRGPRX2 versus FcεRI) and the involved signaling pathways (Gaudenzio et al., 2016; Espinosa and Valitutti, 2018). Furthermore, a detailed spatiotemporal analysis of the dynamics of MC degranulation following exposure to large antigens targeted by IgE/IgG revealed that MC are able to form a unique immunological synapse called antibody-dependent degranulatory synapse (ADDS), which may be relevant to increase the local concentration of inflammatory mediators for example to fight parasite pathogens inactivated by proteases (Joulia et al., 2015; Espinosa and Valitutti, 2018).

### SNARE Proteins

MC degranulation relies on the evolutionary conserved membrane fusion machinery implicating Soluble N-ethyl-maleimide-sensitive factor Attachment protein Receptor (SNARE) proteins (Hong, 2005; Sudhof and Rothman, 2009; Jahn and Fasshauer, 2012). SNARE lie on opposing intracellular membranes and through their SNARE helical motif of about 60 aa can form a stable multimeric complex that catalyzes fusion (Figure 2). A typical SNARE complex at the PM includes a vesicular SNARE (v-SNARE) such as a vesicle associated membrane protein (VAMP) family member that pairs with two target SNARE (t-SNARE) such as synaptosome-associated protein (SNAP) of 23, 25, 29, 47 kDa (SNAP-23/25/29/47) and a Syntaxin (STX) family member (Hong, 2005; Sudhof and Rothman, 2009; Jahn and Fasshauer, 2012). SNAP-23 was the first functional t-SNARE described in MC exocytosis (Figure 2 and Table 1). Introduction of Abs against SNAP-23 into permeabilized rat peritoneal MC (RPMC) blocked stimulus-secretion coupling (Guo et al., 1998). During stimulation SNAP-23 relocated into the interior of the cell forming degranulation channels, a feature compatible with the compound mode of exocytosis. The implication of SNAP-23 in compound exocytosis and its association with SG was found to depend on the endosomal GTPase Rab5 and SNAP-23 phosphorylation on Ser95 and Ser120 mediated by IκB kinase 2/β (IKKβ2) (Klein et al., 2017). Treatment with an inhibitor of IKKβ2 (BMS-345541) switched the degranulation pattern from a compound mode to a unit mode (Gaudenzio et al., 2016). The role of SNAP-23 as an essential SNARE protein was confirmed by others including in mature human MC (Vaidyanathan et al., 2001; Sander et al., 2008; Woska and Gillespie, 2011). SNAP-23 formed complexes with both t-SNARE (STX4, STX3) and v-SNARE (VAMP2, VAMP7, VAMP8) and complexes with VAMP7 and VAMP8 were enhanced after stimulation (Figure 2; Paumet et al., 2000; Sander et al., 2008; Tiwari et al., 2008). Concerning STX family members, siRNA-mediated KD or introduction of an inhibitory peptide of PM-localized STX4 inhibited IgE-mediated degranulation (Woska and Gillespie, 2011; Brochetta et al., 2014; Yang et al., 2018; Table 1 and Figure 2). Like for SNAP-23, STX4 showed an enhanced formation of complexes with VAMP8 after stimulation (Tiwari et al., 2008). The implication of STX4 was recently put into question as MC obtained from STX4 conditional KO mice degranulated normally (Sanchez et al., 2019). However, it is not yet clear whether other STX family members could compensate for the loss of STX4. Indeed, in the same study complete genetic deficiency of STX3 largely blunted degranulation (Sanchez et al., 2019) confirming in this case results from siRNA experiments or experiments with STX3 mutants that indicated an important role of STX3 (Brochetta et al., 2014; Tadokoro et al., 2016; Sanchez et al., 2019; Table 1 and Figure 2). In contrast to STX4, STX3 is mainly located on SG but relocates to the PM upon stimulation, taking the opposite direction to SNAP-23 (Figure 2; Guo et al., 1998; Brochetta et al., 2014; Munoz et al., 2016). This suggests that STX3 could be a component of SNARE complexes both for the fusion between SG but also with the PM, which again is compatible with the compound mode of MC exocytosis. Another t-SNARE, STX11, a lipid anchored t-SNARE, has been proposed to be implicated in MC exocytosis based on its interaction with Munc18-2, a known regulator of MC degranulation (Gutierrez et al., 2018), and its ability to support fusion in other hematopoietic cells (CD8 T and NK cells, neutrophils) (Cote et al., 2009; D’Orlando et al., 2013; Table 1). However, studies with STX11-deficient BMMC did not reveal a MC degranulation defect (D’Orlando et al., 2013).

Concerning v-SNARE, several studies reported a role of VAMP8 (Figure 2 and Table 1). Both native and transduced forms of VAMP8 colocalized with SG in the RBL MC line and primary mature MC (Paumet et al., 2000; Tiwari et al., 2008; Horiguchi et al., 2016). One study (Tiwari et al., 2008) showed that VAMP8-deficient BMMC released less histamine and β-hexosaminidase while cytokine/chemokine secretion was intact. Introduction of soluble recombinant VAMP8 ‘or siRNA-mediated KD in RBL MC also inhibited β-hexosaminidase (Lippert et al., 2007) or β-hexosaminidase and histamine release (Woska and Gillespie, 2011). A study performed in pancreatic acinar cells proposed that VAMP8 may serve only in SG fusion, however, in MC VAMP8 readily gets recruited to the PM in stimulated cells (Tiwari et al., 2008; Horiguchi et al., 2016; Malmersjo et al., 2016; Wilson et al., 2016). Another study reported that VAMP8 deficiency only affects β-hexosaminidase but not histamine release, suggesting that these mediators might be stored in different granule compartments (Puri and Roche, 2008). However, this was not observed by others and is incompatible with data that VAMP8 KO mice also exhibited reduced passive anaphylactic responses *in vivo*, which is dependent on histamine (Tiwari et al., 2008; Woska and Gillespie, 2011). Several other v-SNARE were also analyzed for their role in MC degranulation (Table 1). Introduction of blocking Abs to VAMP7 and its siRNA-mediated KD inhibited secretion, respectively, in primary human MC and RBL MC (Sander et al., 2008; Woska and Gillespie, 2011). VAMP7 translocated to the PM upon stimulation forming enhanced complexes with SNAP-23 and STX4 (Sander et al., 2008; Woska and Gillespie, 2011). Presently, however, no clear localization of VAMP7 to SG has been demonstrated in MC and initial studies, contrary to VAMP8, did not show a colocalization of VAMP7 with the SG compartment in RBL cells (Paumet et al., 2000). Concerning VAMP2 (also called synaptobrevin), initial studies showed that a VAMP2 fluorescent probe transfected into RBL MC readily translocated to the PM upon stimulation (Miesenbock et al., 1998). Some studies showed evidence for a role of VAMP2 in fusion using blocking agents introduced into permeabilized RBL cells (Yang et al., 2018) while others using primary human MC did not (Sander et al., 2008). Importantly, BMMC cultured from VAMP2-KO mice did not show a degranulation defect (Puri and Roche, 2008). Furthermore, localization studies with Abs to endogenous VAMP2 did not reveal colocalization with SG, albeit some interaction with SNAP-23 was detectable (Paumet et al., 2000; Tiwari et al., 2008). While this interaction did not increase after stimulation, it did so in VAMP8-deficient BMMC, opening up the possibility that VAMP2 may replace VAMP8 as a relevant v-SNARE under particular circumstances (Tiwari et al., 2008). Yet another study reported increased association even in the presence of VAMP8 (Suzuki and Verma, 2008). In VAMP8-deficient BMMC VAMP3 also formed enhanced complexes with SNAP-23. However, these complexes decreased upon stimulation, suggesting that VAMP3 may play a role in constitutive fusion mechanisms, which get downregulated during stimulation (Tiwari et al., 2009). In agreement, no evidence for an implication of VAMP3 in MC degranulation was found as VAMP3-deficient BMMC had no defect in SG release (Puri and Roche, 2008). Nonetheless, in macrophages, an important role of VAMP3 was reported in cytokine secretion with VAMP3-containing recycling endosomes serving as an intermediate sorting compartment once cytokines have exited the trans-Golgi (Manderson et al., 2007). However, in MC no effect of VAMP3 inhibition (using blocking Abs) was seen on chemokine secretion while several other SNAREs (STX3, SNAP-23, and for some chemokines VAMP8, STX4, and STX6) were shown to play a role (Frank et al., 2011).

### Regulation of SNARE Complex Formation by Phosphorylation

Although SNARE complexes may support membrane fusion spontaneously (Weber et al., 1998), in living cells this process is highly regulated, implying multiple upstream signaling pathways and accessory proteins (Sudhof and Rothman, 2009). In MC, it has been known for many years that increases in intracellular Ca2+ levels, activation of Ca2+-dependent and independent PKC isoforms (PKCβ, PKCδ), and many other effectors represent essential early signaling intermediates of MC degranulation (Blank and Rivera, 2004; Figures 1, 2). However, the connection to the late signaling events regulating the last steps of the exocytotic process is still incompletely worked out. Phosphorylation of cognate SNARE proteins could represent an important regulatory step. It was reported that murine SNAP-23 was phosphorylated on Ser95 and Ser120 in the cys-rich linker region between its two SNARE domains. Phosphorylated SNAP-23 was enriched in SNARE complexes present in stimulated cells, indicating that this modification directly supports fusion (Hepp et al., 2005; Suzuki and Verma, 2008). In particular, phosphorylation of SNAP-23 may be relevant for compound mode of exocytosis enabling, together with Rab5, its relocation to SG (Klein et al., 2017). Although it was initially reported that SNAP-23 phosphorylation involved PKC (Hepp et al., 2005), later studies reported that this was mediated by IKKβ2 (Figure 2; Suzuki and Verma, 2008). Yet, this was not confirmed in another study, leaving open the possibility of phosphorylation through PKC (Peschke et al., 2014). Concerning STX family members, *in vitro* studies showed that STX4 (but not STX2 and 3) was phosphorylated in its N-terminal regulatory domain by a Rab3D associated kinase (Rak3D), preventing its interaction with SNAP-23 (Figure 2). Phospho-STX4 could be found in living cells. As Rak3D dissociated from Rab3D in a Ca2+-dependent manner, STX4 phosphorylation could be a negative regulator of fusion in resting MC (Pombo et al., 2001). Likewise, STX3 was found to be constantly phosphorylated on Thr14 in the N-terminal peptide domain (Table 1). Mutating this residue enhanced degranulation, indicating a negative regulatory role (Tadokoro et al., 2016). As STX3 phosphorylation inhibited binding to Munc18-2, the inhibitory effect was attributed to its diminished ability to interact with an essential SNARE accessory protein Munc18-2. Concerning v-SNARE family members, it was found that non-neuronal v-SNAREs (e.g., VAMP4, 5, 7, and 8) contain between one to four phosphorylation sites for PKCβ, with VAMP8 containing four (Thr47/53; Ser54/61) located at the interface for SNARE zippering. Phosphomimetic mutants of each individual residue prevented fusion both in *in vitro* liposome fusion assays and in living cells albeit vesicle docking was maintained suggesting that it is the SNARE complex formation that is blocked (Malmersjo et al., 2016).

### Calcium Sensors in MC Fusion

Concerning the role of Ca2+ in MC secretion, several molecular targets of the membrane fusion process have been identified. These include a synaptotagmin (Syt) family member. Syts are transmembrane anchored proteins containing tandem C2 domains (C2A and C2B), which display Ca2+-dependent phospholipid binding (Pinheiro et al., 2016; Brunger et al., 2019). Although initially reported to be a negative regulator in RBL MC (Baram et al., 2002), newer studies using MC from KO animals clearly attributed a positive role of the perigranular localized calcium sensor Syt2 in MC exocytosis (Melicoff et al., 2009; Table 1 and Figure 2). Absence of Syt2 inhibited both histamine and β-hexosaminidase release by close to 70% in BMMC exposed to IgE/Ag. Syt2-KO animals also exhibited a reduced passive cutaneous anaphylaxis responses (PCA) (Melicoff et al., 2009). Likewise, *in vitro* liposome fusion of a MC-relevant SNARE complex (SNAP-23/STX3/VAMP8) was clearly enhanced by the addition of Syt2 and Ca2+ (Nagai et al., 2011). However, little is known about the mechanism of action of Syt2 in MC. Syt action has been mostly worked out for the asynchronous release at the synapse in neuronal cells. In these cells, according to a new “*release of inhibition* model” based on 3D structural data (Brunger et al., 2019), fusion is triggered very fast (within milliseconds) from a primed state where two neuronal Syt1 molecules interact with a preassembled SNARE prefusion complex that also contains complexin (CPLX) (see below). Arrival of Ca2+ dislodges Syt, thereby unlocking the prefusion complex to allow SNARE zippering. This pulls the membranes together, likely assisted by the parallel induced phospholipid binding of Syt1 (and eventually other effectors such as CPLX, Munc13-4, Doc2α; see below) that favor docking and membrane curvature. Together, this fusion machinery may comprise an assembly of a multiprotein complex forming a buttressed ring that acts as a work station for SNAREpin assembly, clamping, and release (Figure 2; Rothman et al., 2017). As MC exocytosis usually takes minutes and not milliseconds, it is presently unclear whether such a priming mechanism also applies to MC exocytosis. Yet, based on the described positive role of Syt2 and CPLX (see below) it is possible that the prefusion complex just represents a short-lived intermediate state during exocytosis. It is also not clear how Syt2, which like Syt1 is a Ca2+ sensor of low affinity (Pinheiro et al., 2016), couples to Ca2+ in MC as the required Ca2+ concentration for secretion is an order of magnitude lower than in neurons (1 versus 20 μM; Blank and Rivera, 2004), although high local concentration at fusion sites may still be relevant.

In this scenario another effector, CPLX, is also relevant as it enables Syt binding to the ternary SNARE complex (Brunger et al., 2019). CPLX is a small (13 kDa) cytoplasmic protein, which exists in several isoforms, with CPLX1 being exclusively neuronal while CPLX2 being expressed in MC lines (RBL, PT18, expression in primary MC has not been investigated) (Table 1). CPLX is composed of short N- and C-terminal sequences that in neurons support, respectively, fast Ca2+-triggered release and membrane lipid binding. It also contains two central α-helices the N-terminal one may regulate neuronal spontaneous exocytosis while the central domain is the one interacting with the preassembled (non-zippered) trans-SNARE complex and Syt (Maximov et al., 2009; Sudhof and Rothman, 2009; Brunger et al., 2019). In RBL MC siRNA-mediated KD of CPLX2 indicated a positive regulatory role in exocytosis. It also translocated from a punctuate cytoplasmic (probably granular) staining pattern to a PM location (Tadokoro et al., 2005). In pulldown experiments CPLX2 preferentially interacted with a complex containing STX3/SNAP-23/VAMP2/8 present in RBL cell lysates, while no STX4 containing complex was detectable (Tadokoro et al., 2010). Together, these results could be in agreement with a role of CPLX2 supporting Syt2 binding and Ca2+ sensitivity of the fusion machinery (Figure 2).

Another important Ca2+ sensor in MC is mammalian uncoordinated 13-4 (Munc13-4) protein, a member of the invertebrate/mammalian Unc13/Munc13s family of proteins involved in vesicle docking and priming (Palfreyman and Jorgensen, 2017; Brunger et al., 2019; Table 1 and Figure 2). Munc13 proteins are large multi-domain proteins. While Munc13-4 shares the two characteristic Munc Homology Domains (MHD) as well as C2A and C2B domains with the neuronal isoforms Munc13-1, 2, and 3, it does not contain the calmodulin and DAG binding sites of other members of this family (Pinheiro et al., 2016; Bin et al., 2018). In neuronal cells the MHD domains are important for the priming function as they allowed the transition of the closed Munc18-1-bound conformation of STX1 to the open conformation involving N-terminal peptide binding, which can engage in SNARE complex formation (Brunger et al., 2019). Munc13-4 was initially described as the mutated effector responsible for familial hemophagocytic lymphohistiocytosis type 3 (FHL3), where patients fail to exocytose docked cytotoxic granules in cytotoxic T cells (Feldmann et al., 2003). Studies in MC using Munc13-4 (or Unc13d or jinx) KO animals showed a severe degranulation defect (Singh et al., 2013; Rodarte et al., 2018). Further functional assessment indicated that in MC, like in T cells, Munc13-4 interacts with SG-localized Rab27a (Neeft et al., 2005; Singh et al., 2013) contributing together with the, respectively, SNARE and phospholipid binding C2A and C2B domains (both of which get activated by Ca2+) (Boswell et al., 2012; Woo et al., 2017; Bin et al., 2018) to the bridging of opposing membranes. This may also support membrane curvature, which in MC may occur both between SG membranes and between SG membrane and the PM (Figure 2; Elstak et al., 2011; Woo et al., 2017). Bridging may further imply a Rab27 binding Slp (PCR data show expression of Slp2 and Slp3 in BMMC; Munoz et al., 2016) molecule as demonstrated in neutrophils and T cells, although this has yet to be shown for MC (Ramadass and Catz, 2016). The corralling of SG beneath the PM, and hence fusion, is lost in cells expressing point mutants of Munc13-4 that do not bind Rab27 (Elstak et al., 2011). As deduced from single molecule FRET studies in liposome fusion assays with neuronal SNAREs, another important function of Munc13-4, executed together with Munc18 proteins, is to control the correct assembly of parallel alpha-helices of the SNARE complex (Lai et al., 2017; Brunger et al., 2019).

Another calcium sensor Doc2α may further participate in this docking and priming process (Table 1). Doc2 are small cytoplasmic proteins, which contain the brain specific isoform DOC2α, and the ubiquitous isoforms Doc2β and Doc2γ (Orita et al., 1997). Doc2 proteins possess an N-terminal Munc13-interacting (Mid) domain and tandem C2A and C2B domains with the C2A domain exhibiting phospholipid binding in a Ca2+-dependent manner (Orita et al., 1996). Strikingly, it was found that MC express the brain specific DOC2α isoform and that MC degranulation was reduced in BMMC from Doc2α-KO mice (Higashio et al., 2008). In RBL MC, Doc2α colocalized with Munc13-4 on SG and interacted with Munc13-4 through its N-terminal Munc13-interacting domain and the C-terminal C2B domain (Higashio et al., 2008). Hence, it was proposed that Doc2α represents an additional Ca2+ sensor assisting to anchor Munc13-4 at the PM to fulfill its priming function (Figure 2; Elstak et al., 2011).

### SM Family Proteins

Sec1/Munc18 (SM) family proteins are crucial effectors in membrane trafficking and exocytosis. Three family members, neuronal Munc18-1, as well as ubiquitously expressed Munc18-2 and Munc18-3, play a role in regulated exocytosis. The predominant isoforms expressed in MC are Munc18-2 and Munc18-3 (Table 1). Munc18 proteins bind to STX t-SNAREs (hence they are also called STXBP1, 2, and 3) with a certain degree of specificity: Munc18-1 preferentially binding to STX1, 2, and 3 (Hata and Südhof, 1995), Munc18-2 to STX1 and 3 (Hata and Südhof, 1995; Martin-Verdeaux et al., 2003), Munc18-3 to STX2 and 4 (Tellam et al., 1995; Martin-Verdeaux et al., 2003). Crystal structure analysis showed that Munc18-1 binds to its STX1 partner in a closed conformation unable to undergo fusion. However, this may not be the case for Munc18-2, which like shown in the structure analysis of Munc18-3 could prefer binding to its STX partner in an open conformation driven by N-peptide binding (Hu et al., 2007; Christie et al., 2012). Independent of these structural considerations, Munc18 proteins are crucial effectors of the fusion process. In animals deficient for Munc18-1 neuronal synaptic transmission was completely abolished (Verhage et al., 2000). Likewise, in MC absence of the STX3-binding, Munc18-2 strongly inhibited homo- and hetero-typic fusion both in BMMC and in *ex vivo* derived RPMC (Gutierrez et al., 2018) confirming earlier results obtained in RBL cells or BMMC using KD experiments (Tadokoro et al., 2007; Bin et al., 2013; Brochetta et al., 2014). This concurs also with results indicating that STX3 is a major t-SNARE in MC exocytosis. Munc18-2 KD also affects secretion in other hematopoietic cells, being responsible for the secretory defect in FLH5 patients carrying different mutations (Cote et al., 2009; Cardenas et al., 2019). By contrast, no roles for Munc18-1 and Munc18-3 could be delineated in MC, although Munc18-3 seemed to play a role in neutrophil exocytosis (Martin-Verdeaux et al., 2003; Brochetta et al., 2008; Gutierrez et al., 2018). It is likely that mechanistically Munc18-2 plays a similar role than the neuronal isoform Munc18-1 in membrane fusion as already described above (Brunger et al., 2019). However it is possible that Munc18-2 may already in unstimulated cells bind in an open fusion-competent conformation to a single STX3 molecule as reported for Munc18-3 and STX4 (Hu et al., 2007; Christie et al., 2012) prior to the stimulation-induced binding to the assembled SNARE complex (Figure 2). Both Munc18-2 and Munc13-4 act then together as assembly factors in a coordinated fashion to enable proper structural alignment of a primed and Syt bound ternary SNARE complex (Figure 2; Brunger et al., 2019). Contrary to neurons, where vesicles need to be ready to fuse within milliseconds, the primed complex in MC may represent a short-lived intermediate state.

Biochemical studies in MC showed a differential STX3 and Munc18-2 distribution into rafts (Pombo et al., 2003). Likewise, functional data supported additional effects on exocytosis (Brochetta et al., 2014), suggesting that Munc18-2, besides assembling the fusion complex, might have supplementary functions. These could relate to a functional SG docking. In chromaffin cells a docking defect of SG at the PM was observed in the absence of neuronal Munc18-1, a property that may depend on its ability to interact with Rab3a (Voets et al., 2001; van Weering et al., 2007). Likewise, a Munc18-1-dependent docking for SG in insulin secreting cells with stable docking being promoted by formation of clusters of STX1/Munc18-1 at the nascent granule docking site being further supported by Rab3a known to be expressed on SG and able to interact with Munc18 (Gandasi and Barg, 2014). Indeed, interactions of Rab3 proteins with Munc18 isoforms have been proposed before to be relevant for the fusion process, but this awaits further studies in MC (Graham et al., 2008; Gandasi and Barg, 2014). Based on this it seems possible that besides its role in fusion, Munc18-2, like Munc18-1, may also dynamically regulate docking, which, in the case of Munc18-2, may also involve docking at the microtubule cytoskeleton. Munc18 proteins are also known to represent a downstream target of kinases. Thus, tyrosine phosphorylation by neuronal Src family kinases (SFK) of Munc18-1 at Y473 (a site conserved in Munc18-2) prevents its ability to promote fusion and was proposed as a powerful mechanism to block synaptic transmission (Meijer et al., 2018). Likewise, PKC- and CDK5-dependent phosphorylation favored, respectively, vesicle pool replenishment in neurons and enhanced insulin secretion in pancreatic β cells (Lilja et al., 2004; Nili et al., 2006). However, a regulatory role of phosphorylation of Munc18-2 has not yet been reported in MC.

### SCAMP

Another family of fusion accessory proteins are secretory carrier membrane proteins (SCAMP). These are tetraspanins with major isoforms expressed in MC being SCAMP1 and SCAMP2 (Table 1). Both SCAMP 1 and 2 in MC localize to SG, but a small fraction is also found at the PM where they co-localize with STX4 and SNAP-23 (Figure 2). Both isoforms can also be co-immunoprecipitated with SNAP-23. Expression of a peptide (E-peptide), within the second and third transmembrane (TM) domain, potently inhibits exocytosis in permeabilized RPMC (Guo et al., 2002) with the peptide derived from SCAMP2 being an order of magnitude more potent than SCAMP1, suggesting that this isoform represents the major regulator of the fusion process (Table 1). SCAMP2 may act at a late step that couples Arf6-stimulated phospholipase D (PLD) activity to the formation of fusion pores. PLD catalyzes the hydrolysis of phosphatidylcholine to generate the lipid second messenger, phosphatidate (PA), important in the regulation of the membrane phospholipid PIP2 that is important at fusion sites. This could be in agreement with the proposed implication of PLD isozymes (granular localized PLD1 and PM localized PLD2) as regulators of MC exocytosis (Figure 2; Cockcroft et al., 2002).

### Tomosyn-1 (STXBP5)

Tomosyn is encoded by two related genes, tomosyn-1 (STXBP5) and tomosyn-2 (STXBP5L). MC express high amounts of tomosyn-1 mRNA encoding a large (∼130 kDa) protein with a C-terminal v-SNARE domain and 14 N-terminal tryptophan-aspartic acid (WD) 40 repeats forming propeller-like structures as protein interaction platforms (Hattendorf et al., 2007; Table 1). Both domains are reported to participate in inhibiting vesicle membrane fusion (Pobbati et al., 2004; Bielopolski et al., 2014) either by preventing access to the cognate v-SNARE or by promoting fusion-incompetent ternary SNARE complex oligomerization. Studies in different cells showed that tomosyn binds STX and inhibits secretory events including in non-neuronal cells where it restricts insulin secretion (Zhang et al., 2006) and endothelial cell exocytosis (Zhu et al., 2014). However, positive regulatory actions have been also reported in pancreatic β cells, platelets, and in yeast expressing the homologue Sro7, the latter of which, however, lacks the C-terminal v-SNARE domain (Cheviet et al., 2006; Hattendorf et al., 2007; Ye et al., 2014). A positive regulatory role was also attributed to a C-terminal tail region preceding the tomosyn v-SNARE domain that upon interaction with its STX partner may then serve as a facilitator for cognate v-SNARE interaction (Yamamoto et al., 2010). This may suggest that tomosyn has more complex roles. Studies in MC showed that tomosyn-1/STXBP5 acts as a fusion clamp as specific KD enhances degranulation (Madera-Salcedo et al., 2018). Further analysis revealed that it binds to STX4 in resting cells but dissociates after stimulation, which could explain the fusion clamp mechanism (Figure 2). Dissociation was mediated by stimulation-induced Ser and Thr phosphorylation, implicating the Ca2+-independent PKCδ isoform as KD of the latter prevented dissociation. Increased phosphorylation was notably observed in the regulatory second loop region and tail region preceding the v-SNARE domain (Yamamoto et al., 2009; Madera-Salcedo et al., 2018). Strikingly, the situation was different for STX3 as stimulation rather induced its phosphorylation-dependent association with tomosyn-1/STXBP5. Although the functional significance of the increased association with STX3 remains unclear, it was proposed that this STX binding switch may act as a feedback mechanism at the membrane once STX3 gets translocated to gradually block membrane fusion to limit eventually life-threatening anaphylactic reaction due to excessive MC activation (Madera-Salcedo et al., 2018).

### Rab Proteins

Among the sixty Rab expressed in mammals, several of them have been already mentioned above as they regulate important trafficking steps, coupling granule trafficking to the microtubule and actin cytoskeleton (Figure 1). We will only discuss briefly the implication of those that may directly be implicated in the membrane fusion process. One candidate is Rab3D found to be the most prevalent Rab3 isoform expressed in MC (Roa et al., 1997; Tuvim et al., 1999; Table 1 and Figure 2). It partially localized to SG in RBL MC, but fully in PMC and transiently translocated to the PM upon exocytosis. Overexpression of a constitutively active mutant blocked secretion (Roa et al., 1997; Tuvim et al., 1999). A MC effect was not confirmed in Rab3D-KO mice, although these mice exhibited increases in the size of SG in both the exocrine pancreas and the parotid gland but not in MC, which suggested a role in SG maturation (Riedel et al., 2002). However, potential compensatory mechanisms by other isoforms (Rab3A, Rab3B, and Rab3C) have not been investigated in this study (Riedel et al., 2002). Rab37 was also found highly expressed in BMMC and able to interact in a GTP-independent manner with Munc13-4 in a trimeric complex containing Rab27 and Munc13-4 (Masuda et al., 2000; Higashio et al., 2016; Table 1 and Figure 2). This trimeric complex formation (Rab37-Munc13-4-Rab27) negatively regulates MC degranulation through a probable inhibition of the SG docking/priming step with the PM (Higashio et al., 2016). Rab5 may also play an important role in the fusion process favoring compound/multigranular exocytosis (Table 1). Indeed, based on studies with constitutively active mutant forms and KD experiments, Rab5 was attributed a key role in SG maturation mediating SG fusion during biogenesis controlling the amount and composition of the SG content (Azouz et al., 2014). Additional studies then showed that Rab5 also directly regulates SNAP-23-mediated SG-SG fusion during stimulation.

The above data clearly show that in living cells the fusion process is regulated, besides cognate SNARE proteins, by a multitude of molecular effectors that can be connected to upstream signals such as Ca2+ influx and phosphorylation and which may vary from cell type to cell type. It becomes thus clear that this multitude of molecular actors further increases the complexity of the molecular interactions and regulatory steps to be worked out.

## Concluding Remarks

MC signaling dowstream the FcεRI stimulation has been widely studied during the last 20 years. However, it is not entirely clear how the molecular processes governing the cytoskeleton reorganization and the SG translocation and fusion are connected to upstream signaling pathways upon MC activation. Decoding new effectors involved in their interplay will unveil new knowledge into MC biology. This field of research will also benefit from the advances that will be made in other cell types of immune or non-immune origin, which are capable of regulated secretion and share the same molecular machineries that regulate cargos translocation and fusion. In addition to FcεRI, MC express many other receptors (Toll-like receptors, complement receptors, neuropeptide and neurotransmitter receptors, lipid mediator receptors, etc.) able to respond to a highly diverse array of products (including neuropeptides, complement fragments, cationic compounds, environmental substances, and other inflammatory mediators) and trigger SG release (Redegeld et al., 2018). While these receptors contribute to the sentinel function of MC, they may also cause their inappropriate activation in an inflammatory setting, thereby participating to chronic activation, for example, during the allergic response. Hence, it will be important to further progress in the understanding of the crosstalk between signaling pathways and MC degranulation to delineate all involved effector mechanisms to design novel therapeutic approaches for controlling allergic reactions.

## Author Contributions

GM and UB contributed equally in writing and editing the manuscript. GM, CL, MB, and UB created the figures. All authors corrected the manuscript and approved the submitted version.

## Conflict of Interest

The authors declare that the research was conducted in the absence of any commercial or financial relationships that could be construed as a potential conflict of interest.

## Glossary

ADDS, Antibody-dependent degranulatory synapse; APC, Adenomatous polyposis coli; Arp2/3, Actin-related proteins 2/3; BMMC, Bone marrow-derived MC; CPLX, Complexin; CRAC, Ca2+ release activated channel; DAG, Diacylglycerol; DBN, Drebrin; EB1, End-binding protein 1; ER, Endoplasmic reticulum; ERM, Ezrin Radixin Moesin; FEZ1, Fasciculation and elongation protein zeta 1; FHL, Familial hemophagocytic lymphohistiocytosis; Gab2, Grb2-associated binding protein 2; Gads, Grb2-related adaptor protein; GCPs, γ-tubulin complex proteins; GEF, Guanine nucleotide exchange factor; GIMP, Gem-interacting protein; GIT1, G protein-coupled receptor kinase-interacting protein 1; Grb2, Growth-factor-receptor bound protein 2; IKKβ2, IκB kinase 2/β; IP3, Inositol 1,4,5-trisphosphate; ITAM, Immunoreceptor tyrosine-based activation motifs; KD, Knockdown; KO, Genetic knockout; LAT, Linker for Activation of T cells; LIMK, LIM-motif containing kinase; MAPs, Microtubule-associated proteins; MC, Mast cells; mDia1, Mammalian diaphanous-related formin 1; Mid, Munc13-interacting domain; MHD, Munc Homology Domains; MLCK, Myosin light chain kinase; MLCP, Myosin light chain phosphatase; MTOC, Microtubule Organizing Center; Munc13-4, Mammalian uncoordinated 13-4; ORAI1, Ca2+ release-activated Ca2+ channel protein1; PA, phosphatidate; PAK, p21-activated kinase; PCA, Passive cutaneous anaphylaxis responses; PI3K, Phosphatidyl inositol-3 kinase; PIP3, Phosphatidylinositol (3,4,5)-trisphosphate; βPIX, p21-activated kinase interacting exchange factor β; PKC, Protein kinase C; PLCγ, Phospholipase Cγ; PLD, Phospholipase D; PM, Plasma membrane; PMD, Piecemeal degranulation; PP2A, Protein phosphatase 2; PTK, Protein tyrosine kinases; RasGRP1, Ras guanyl nucleotide-releasing protein 1; RBL, Rrat basophilic leukemia; RE, Recycling endosomes; RhoA, Ras homology family member A; RILP, Rab-interacting lysosomal protein; ROCK, Rho-associated coiled-coil kinase; RPMC, Rat peritoneal MC; SCAMP2, Secretory carrier membrane protein 2; SFK, Src family kinases; SG, Secretory granules; SHP-1, Src homology 2 domain-containing protein tyrosine phosphatase 1; SLP-76, SH2 domain-containing leukocyte phosphoprotein of 76 kDa; SM, Sec1/Munc18; SNAP, Synaptosome-associated protein; SNARE, Soluble N-ethyl-maleimide-sensitive factor Attachment protein Receptor; SOCs, Store-operated channels; STIM-1, Stromal interaction molecule 1; STX, Syntaxin; STXBP, Syntaxin binding protein; Syt, Synaptotagmin; TM, Transmembrane; +Tips, Microtubules plus-end tracking protein; TRPC1, Transient potential Ca2+ channel 1; γ-TuRC, γ-tubulin ring complex; VAMP, Vesicle associated membrane protein; WASP, Wiskott-Aldrich syndrome protein; WAVE, Wiskott-Aldrich syndrome protein-family verprolin homologous protein; WIP, Wiskott-Aldrich syndrome protein interacting protein.
